# Protein tyrosine phosphatase 1B as a therapeutic target for Graves’ orbitopathy in an *in vitro* model

**DOI:** 10.1371/journal.pone.0237015

**Published:** 2020-08-06

**Authors:** Hyeong Ju Byeon, Ji-Young Kim, JaeSang Ko, Eun Jig Lee, Kikkawa Don, Jin Sook Yoon

**Affiliations:** 1 Department of Ophthalmology, Severance Hospital, Institute of Vision Research, Yonsei University College of Medicine, Seoul, Korea; 2 Department of Internal Medicine, Severance Hospital, Institute of Endocrine Research, Yonsei University College of Medicine, Seoul, Korea; 3 Division of Oculofacial Plastic and Reconstructive Surgery, University of California San Diego, La Jolla, California, United States of America; National Institutes of Health, UNITED STATES

## Abstract

Graves’ orbitopathy (GO) is characterised in early stages by orbital fibroblast inflammation, which can be aggravated by oxidative stress and often leads to fibrosis. Protein tyrosine protein 1B (PTP1B) is a regulator of inflammation and a therapeutic target in diabetes. We investigated the role of PTP1B in the GO mechanism using orbital fibroblasts from GO and healthy non-GO subjects. After 24 hours of transfection with *PTPN1* siRNA, the fibroblasts were exposed to interleukin (IL)-1β, cigarette smoke extract (CSE), H_2_O_2_, and transforming growth factor (TGF)-β stimulations. Inflammatory cytokines and fibrosis-related proteins were analysed using western blotting and/or enzyme-linked immunosorbent assay (ELISA). Reactive oxygen species (ROS) release was detected using an oxidant-sensitive fluorescent probe. IL-1β, tumor necrosis factor (TNF)-α, bovine thyroid stimulating hormone (bTSH), high-affinity human stimulatory monoclonal antibody of TSH receptor (M22), and insulin-like growth factor-1 (IGF-1) significantly increased PTP1B protein production in GO and non-GO fibroblasts. *PTPN1* silencing significantly blocked IL-1β-induced inflammatory cytokine production, CSE- and H_2_O_2_-induced ROS synthesis, and TGF-β-induced expression of collagen Iα, α-smooth muscle actin (SMA), and fibronectin in GO fibroblasts. Silencing *PTPN1* also decreased phosphorylation levels of Akt, p38, and c-Jun N-terminal kinase (JNK) and endoplasmic reticulum (ER)-stress response proteins in GO cells. PTP1B may be a potential therapeutic target of anti-inflammatory, anti-oxidant and anti-fibrotic treatment of GO.

## Introduction

Graves’ orbitopathy (GO) is an orbital involvement of autoimmune thyroid disease; it occurs in approximately 50% of Grave’s disease and 2% of chronic thyroiditis [1, 2]. Clinical manifestations include lid swelling, lid retraction, proptosis, and ocular movement limitations; 3–5% of patients have a severe form, including pain, inflammation, and compressive optic neuropathy [1–3]. Orbital fibroblasts dysregulated by autoantibodies to thyrotropin receptor and IGF-1 receptor (IGF-1R) play a key role in the pathogenesis of GO. Furthermore, orbital fibroblasts differentiate into adipocytes, accumulating fatty tissue and producing hyaluronan, which leads to the typical findings of GO [3, 4]. Mononuclear cells, such as macrophages, T cells, and B cells, infiltrating the orbital tissue orchestrate the pathogenic inflammatory processes; moreover, ROS have been found to aggravate GO [5]. As inflammation in the orbital tissue is the primary aetiology of GO, glucocorticoids have been used as first-line treatment; however, their systemic complications have prompted researchers to explore alternative treatment options.

Protein tyrosine phosphatase 1B (PTP1B), encoded by the *PTPN1* gene, is a ubiquitously expressed non-receptor protein tyrosine phosphatase enzyme that dephosphorylates tyrosine-phosphorylated proteins. It is expressed in multiple tissues, including the skeletal muscle, liver, adipose tissue, and brain [6]. PTP1B is a negative regulator of the leptin and insulin signalling pathways. It has been investigated as a therapeutic agent in diabetes and obesity following observations that mice with whole-body deletion of PTP1B exhibited increased insulin sensitivity and reduced weight gain when reared on a high-fat diet [7, 8]. PTP1B is also known to be involved in immune cell signalling [9, 10] by regulating cytokines via dephosphorylation of janus kinase (JAK)2, signal transducer and activator of transcription (STAT)5, and tyrosine kinase (TYK)2 [11, 12]. However, depending on the cell or tissue type, PTP1B displays different specific roles; for example, PTP1B knockout (KO) induced no change in inflammation in neuronal cells but resulted in upregulation of inflammation in B cells [13].

As the role of PTP1B in GO has not yet been studied, we characterised the effect of PTP1B in Graves’ orbital fibroblasts in response to proinflammatory and oxidative stress challenges, which simulate the main pathogenesis of GO.

## Materials and methods

### Reagents and chemicals

Dulbecco’s modified Eagle’s medium (DMEM), fetal bovine serum (FBS), penicillin, and gentamycin were purchased from Hyclone Laboratories, Inc. (Logan, UT, USA). Recombinant human IL-1β, TNF-α and IGF-1 were purchased from R&D Systems (Minneapolis, UT, USA). M22 was purchased from RSR Ltd. (Llanishen, Cardiff, UK) and bTSH was obtained from Genzyme Corporation (Cambridge, MA, USA). TGF-β and antibodies for PTP1B, phosphorylated (p)-Akt, total (t)-Akt, p-extracellular signal-related kinase (ERK), t-ERK, p-p38, t-p38, p-JNK, t-JNK, cyclooxygenase (Cox)-2, intercellular adhesion molecule (ICAM)-1, p-nuclear factor (NF)-κB, t-NF-κB, binding immunoglobulin protein (BiP), p-eukaryotic translation initiation factor (eIF)2α, t-eIF2α, p-inositol-requiring enzyme (IRE)1α, t-IRE1α, p-protein kinase R-like endoplasmic reticulum kinase (PERK), t-PERK, activating transcription factor (ATF)4, ATF6α, and C/EBP-homologous protein (CHOP) were obtained from Cell Signaling Technology (Beverly, MA, USA), the antibody for fibronectin was obtained from BD Bioscience (Franklin Lake, NJ, USA), and the α-SMA antibody was obtained from Sigma-Aldrich (Saint Louis, MO, USA). collagen Iα, and anti–β-actin antibodies were purchased from Santa Cruz Biotechnology (Santa Cruz, CA, USA).

### Tissue and cell preparation

Orbital adipose tissues were harvested from surgical by-products during orbital decompression surgery in 4 patients with GO (3 males, 1 female; age 37–55 years). At the time of the surgery, patients were in a stable euthyroid state and their clinical activity scores (CAS) were 3 or less. Normal control adipose tissues were obtained from 3 patients with no history of GO (1 male, 2 females; age 36–52 years) during lid blepharoplasty (Table 1). All patients provided written informed consent. This study was approved by the institutional review board of Severance Hospital, Yonsei University College of Medicine (Seoul, Korea) and followed the tenets of the Declaration of Helsinki.

**Table 1:** Clinical information of patient samples used in this *in vitro* study.

Age (years)	Sex	CAS	Smoker	Duration of GO (years)	Proptosis R/L (mm)	Surgery performed
**GO patients**						
37	F	3/7	N	2.0	21/21	Decompression
42	M	1/7	Y	2.1	21/21	Decompression
52	M	0/7	N	4.2	21/20	Decompression
55	M	1/7	N	3.0	23/23	Decompression
**Non-GO control subjects**						
52	F	n/a	N	n/a	n/a	Lower lid blepharoplasty
36	M	n/a	N	n/a	n/a	Upper lid blepharoplasty
51	F	n/a	N	n/a	n/a	Upper lid blepharoplasty

Abbreviations: GO, Graves’ orbitopathy; CAS, clinical activity scores; Y, yes; N, No; n/a; not applicable; F, female; M, male; R, right eye; L, left eye.

Orbital fibroblasts were cultured according to the method used in our previous study [14]. Briefly, the tissue was minced and placed in DMEM:F12 (1:1) containing 20% FBS, penicillin (100 U/mL), and gentamicin (20 μg/mL). Following fibroblast growth, the cells were passaged serially treating them with trypsin/ethylenediaminetetraacetic acid (EDTA). Strains were stored in liquid nitrogen. Only strains between the 3^rd^ and 6^th^ passages were used for experiments.

### Silencing of PTP1B

Small interfering RNA (siRNA) designed to silence the PTP1B gene (*PTPN1*) and negative control #1 siRNA were purchased from Ambion/Applied Biosystems (s11507; Ambion, Austin, Texas, USA). Transfection was performed with Lipofectamine RNAiMAX (Invitrogen) according to the instructions of the supplier. Cells (1 × 10^5^ cells/well) were supplemented with 10% FBS and antibiotics and maintained for a 24-hour period.

### Western blot

Orbital fibroblasts treated with each reagent were washed with phosphate-buffered saline (PBS) and lysed in cell lysis buffer on ice in accordance with the methods described in our previous study [14]. Cell lysates were centrifuged to produce homogenous cell fractions, which were then boiled in buffer. Proteins were resolved by 10% SDS-PAGE, transferred to polyvinylidene fluoride membranes (Immobilon; Millipore Corp., Billerica, MA, USA), and treated with primary antibodies overnight in Tris-buffered saline and Tween 20 (TBST). Immunoreactive bands were detected with horseradish peroxidase and its secondary antibody. The bound peroxidase was visualised by chemiluminescence (Amersham Pharmacia Biotech, Inc., Piscataway, NJ, USA) and exposure to X-ray film (Amersham Pharmacia Biotech, Inc.). Protein was quantified via densitometry and normalised to the level of β-actin in each sample.

### Enzyme-linked immunosorbent assay

To quantify the levels of IL-6, IL-8, and monocyte chemoattractant protein-1 (MCP-1), supernatants collected from cell culture were evaluated using an ELISA kit (R&D system, Minneapolis, UT, USA) according to the manufacturer’s protocol. Absorbance of the samples was measured at 450 nm using microplate reader (Molecular Devices, Sunnyvale, CA, USA) and the percentage of binding was calculated.

### Real-time PCR

RNA was extracted from orbital tissues, which were homogenized with a tissue homogenizer (Precellys^®^ 24; Bertin Instruments, Montigny-le-Bretonneux, France) using a Precellys lysing kit (Bertin Instruments) with TriZol (Invitrogen, Carlsbad, CA, USA) and cDNA was synthesized. PCR was performed using TaqMan universal PCR master mix in an ABI 7300 real-time PCR thermocycler (Applied Biosystems, Carlsbad, CA, USA). Primers for Toll-like receptor (TLR) 2, 4, and 9 were as follows: TLR2; 5’-GAC TTC TCC CAT TTC CGT CT-3’ (forward), 5’-CAG GTA GGT CTT GGT GTT CA-3’ (reverse), TLR4; 5’-GAC CTT TCC AGC AAC AAG ATT C-3’ (forward), 5’-GAG AGA TTG AGT AGG GGC ATT T-3’ (reverse), TLR9; 5’-GGC TGG TAT AAA AAT CTT ACT TCC TC-3’ (forward), 5’-CAC ACT CGA GGT CCC TTC C-3’ (reverse); these primers were designed using Applied Biosystems software and GAPDH were No. Hs99999905_m1. The results were expressed using the 2^−ΔΔCt^ method, as relative exponential changes of threshold cycle (Ct) relative to the control group. The amplified band was quantified through densitometry and normalized against GAPDH. RT-PCR was performed in duplicate in three cells from different individuals.

### Intracellular ROS measurement

CSE and H_2_O_2_ were used to induce ROS production as previously reported [15]. CSE was prepared from two commercially available cigarettes containing 8.0 mg tar and 0.7 mg nicotine (Marlboro 20 class A; Philip Morris Korea, Inc., Seoul, Korea) as described previously [15]. Here, 2% CSE and 200 μM H_2_O_2_ were used to generate oxidative stress in cells without reducing cell viability. Cells (1 × 10^5^ cells/well) were exposed to oxidative stressors, washed with PBS, and incubated with the oxidant-sensitive fluorescent probe 5-(and 6)-carboxy-2’,7’-dichloro-dihydrofluorescein diacetate (H_2_DCFDA; Invitrogen, Eugene, OR, USA) for 30 minutes. The cells were then trypsinised, washed, and resuspended in PBS. To measure fluorescence intensity, a flow cytometer (ELITE flow cytometer; Coulter Cytometry, Inc., Hialeah, FL, USA) was used, and fluorescent photos were taken using an IX71-F22PH inverted fluorescence microscope (Olympus Corp., Tokyo, Japan).

### Cell viability and apoptosis assays

Cell viability after *PTPN1* blockade was evaluated with a 3-(4, 5-dimethyl thiazol-2-yl)-2, 5-diphenyl tetrazolium bromide (MTT) assay. Orbital fibroblasts from GO and non-GO patients with sicontrol or siPTPN1 transfection were seeded in 24-well culture plates (1 × 10^5^ cells/well) and were treated with IL-1β (10 ng/mL) for 24 and 48 hours. After treatment, cells were washed and incubated with 5 mg/mL MTT solution for 3 hours at 37 °C and then stabilized in ice-cold isopropanol. Microplate reader (EL 340 Bio Kinetics Reader; Bio-Tek Instruments, Winooski, VT, USA) was used to measure the absorbance of the dye at 490 nm.

An annexing V-FITC kit (R&D systems, Minneapolis, MN, USA) was used to assess apoptotic cells with *PTPN1* blockade. Cells were washed with PBS and incubated with annexin V labeled with FITC and propidium iodide (PI) for 15 minutes at room temperature. Apoptotic cells were analyzed using a fluorescence-activated cell sorter (FACS, Becton Dickinson-FACScalibur, Cockeysville, MD, USA). In total, 10,000 cells were excited at 488 nm and emission was measured at 515–545 nm and 600 nm to assess FITC and PI, respectively.

### Statistical analysis

IBM SPSS Statistics for Windows v 20.0 (IBM Corp., Armonk, NY) was used for statistical analyses. All experiments were performed in duplicate on three samples from different individuals, and the results are expressed as the mean values ± standard deviation. The Mann–Whitney U-test and Kruskal–Wallis test were used for nonparametric data, and the Kolmogorov–Smirnov test was used for data that was not normally distributed. A *p-*value less than 0.05 was considered statistically significant.

## Results

### TSH and IGF-1 increased PTP1B production in orbital fibroblasts

**Fig 1:**
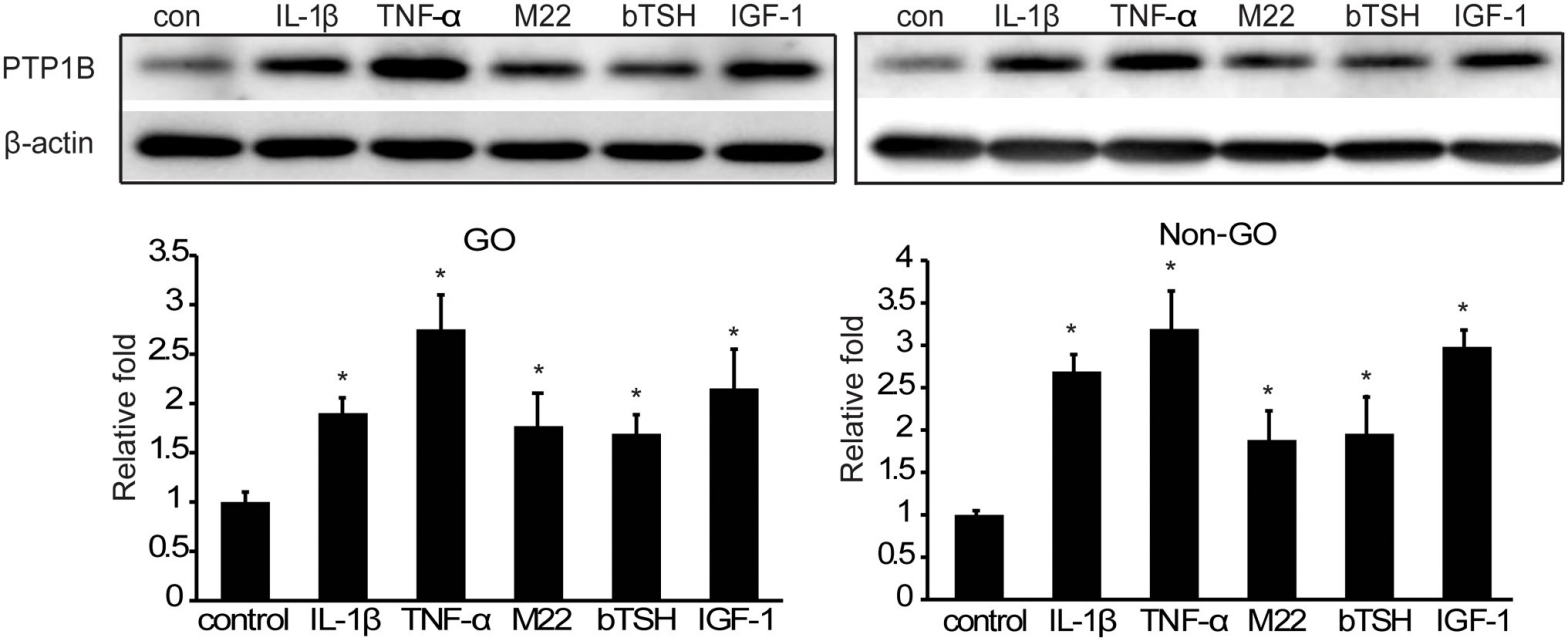
PTP1B protein expression stimulated by proinflammatory cytokines, TSH Receptor (TSHR), and IGF-1R ligands. Orbital fibroblasts cultured from GO and non-GO tissues were treated with IL-1β (10 ng/mL), TNF-α (10 ng/mL), M22 (10 ng/mL), bTSH (2.5 mU/mL), or IGF-1 (100 ng/mL) for 48 hours. Cell lysates were subjected to western blot. IL-1β, TNF-α, M22, bTSH, and IGF-1 significantly increased the expression of PTP1B protein in both GO and non-GO fibroblasts. Experiments were performed in three GO and non-GO cells from different individuals. Data in the columns indicate the mean density ratio ± SD by densitometry normalised to the level of β-actin in the same sample. Representative gel images are shown. Differences between treated and untreated cells are indicated (*p<0.05). See S3 Fig for uncropped blots.

Fibroblasts were stimulated with IL-1β (10 ng/mL) or TNF-α (10 ng/mL) for 48 hours to evaluate PTP1B expression changes in response to inflammatory stimulation in GO and non-GO cells, as previously reported [14, 16, 17]. We also treated cultures with IGF-1 (100 ng/mL), M22 (10 ng/mL), or bTSH (2.5 mU/mL). PTP1B protein production was increased significantly by all these stimulants in both GO and non-GO cells (*p*<0.05) (Fig 1).

### PTP1B inhibition reduces IL-1β-induced inflammation in GO orbital fibroblasts

**Fig 2:**
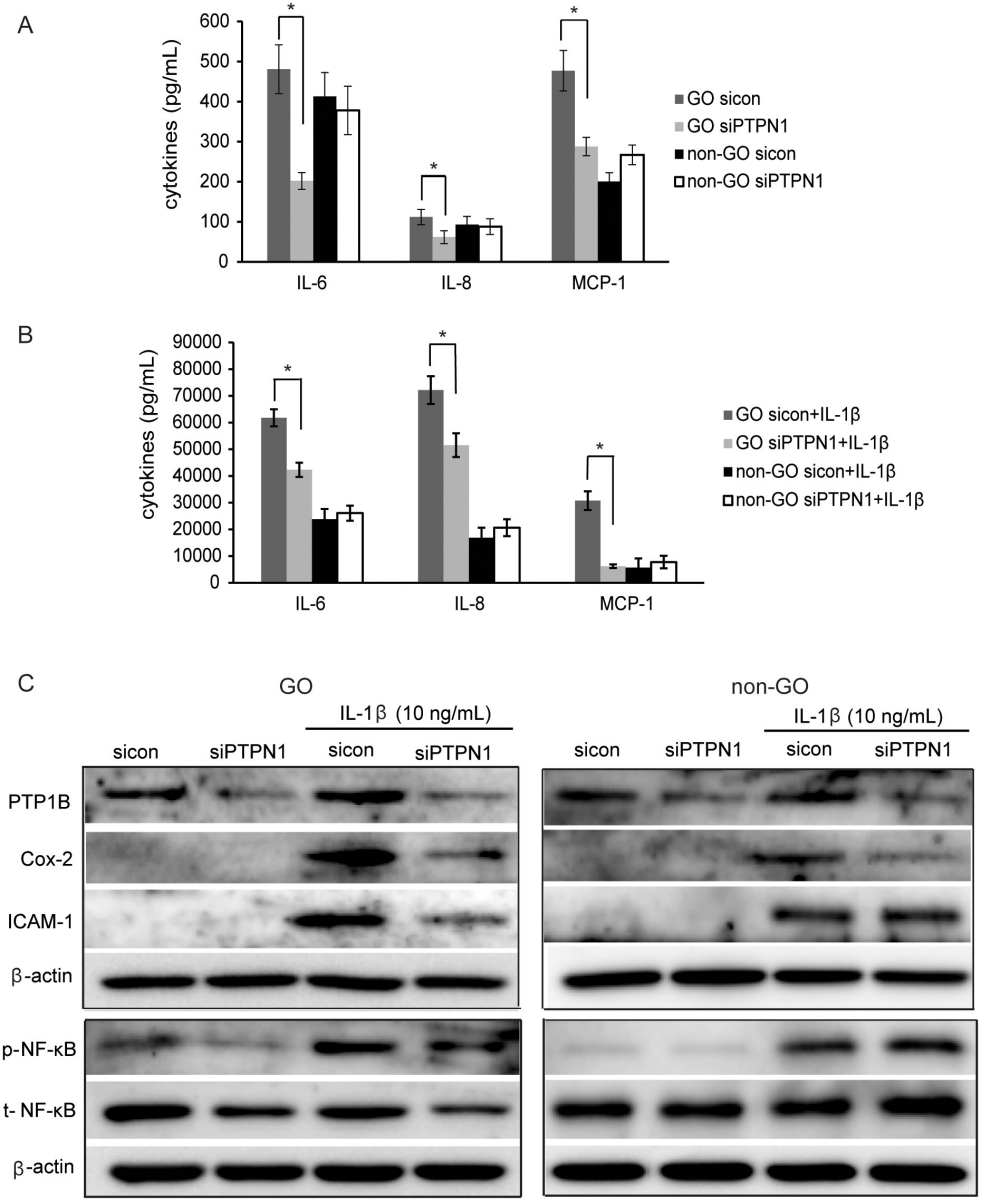
Effect of PTP1B suppression on proinflammatory cytokine production. After transfection of GO and non-GO fibroblasts with siPTPN1 for 24 hours, cells were treated with IL-1β (10 ng/mL) for 1 hour or 48 hours for the evaluation of NF-κB or inflammatory cytokine production, respectively. Experiments were performed in three GO and non-GO cells from different individuals. In sicontrol cells and siPTPN1 cells, IL-6, IL-8, and MCP-1 protein release was analysed under basal condition (A) and after 48 hours of IL-1β (10 ng/mL) stimulation (B) using ELISA (*p<0.05, sicontrol versus siPTPN1 cells). (C) Protein expression levels of ICAM-1, Cox-2, and p-NF-κB in sicontrol and siPTPN1 cells with or without IL-1β (10 ng/mL) stimulation were evaluated using western blot analysis. Representative bands are shown. See S3 Fig for uncropped blots.

To clarify the role of PTP1B in inflammatory stimulation, *PTPN1* was silenced via transfection of *PTPN1*-targeting siRNA (siPTPN1); silencing efficiency of siPTPN1 compared with sicontrol (sicon in figures) was verified using western blot. We then treated *PTPN1*-silenced GO and non-GO cells with IL-1β (10 ng/mL) for 48 hours and measured the production of pro-inflammatory cytokines, IL-6, IL-8 and MCP-1 using ELISA. GO cells secreted more proteins of IL-6, IL-8 and MCP-1 than non-GO cells under basal and IL-1β stimulated condition. Down-regulation of PTP1B by siPTPN1 inhibited the secretion of IL-6, IL-8, and MCP-1 in GO cells with or without IL-1β (*p<0.05), but not in non-GO cells (Fig 2A and 2B). In western blot analyses, IL-1β-induced Cox-2 and ICAM-1 protein production was reduced in siPTPN1-treated GO cells and only Cox-2 expression was attenuated in siPTPN1-treated non-GO cells (Fig 2C). Stimulation of fibroblasts with IL-1β (10 ng/mL) for 1 hour increased the p-NF-κB production; however, this upregulation was suppressed by *PTPN1* silencing in GO cells.

TLRs participate in autoimmune diseases [18] and TLR-related signaling activates NF-κB to secrete inflammatory cytokines [19]. After transfection of GO and non-GO fibroblasts with siPTPN1 for 24 hours, TLR2, 4, and 9 mRNA expression levels were identified through RT-PCR analysis. TLR2, 4, and 9 were suppressed through *PTPN1* inhibition in GO fibroblasts. In non-GO fibroblasts, TLR4 and 9 were suppressed through *PTPN1* inhibition (*p<0.05) (S1 Fig).

### PTP1B inhibition reduces ROS generation upon CSE and H_2_O_2_ stimulation

**Fig 3:**
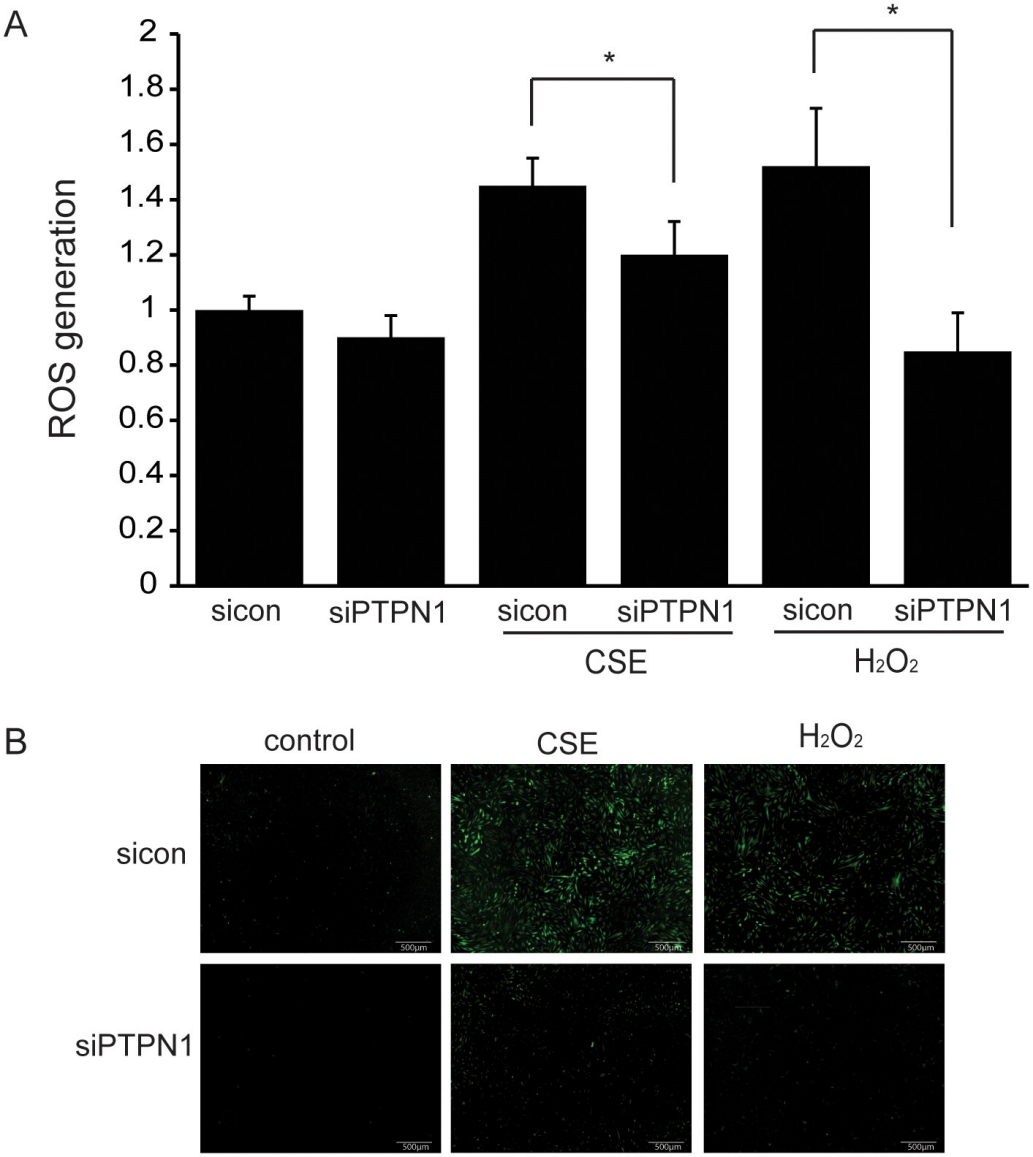
Effect of PTP1B suppression on CSE- or H_2_O_2_-induced intracellular ROS generation. GO cells were transfected with sicontrol or siPTPN1 for 24 hours. Cells were pretreated with H_2_DCFDA for 30 minutes in all GO fibroblast groups. Then, cells were exposed to 2% CSE or 200 μM H_2_O_2_ for 30 minutes. Experiments were performed using three cells from different individual samples. (A) Intracellular ROS generation was quantified by flow-cytometry with H_2_DCFDA. The bar graphs show mean ratios ± SD relative to the sicontrol without CSE or H_2_O_2_ treatment. (*p<0.05, sicontrol versus siPTPN1) (B) ROS production was observed using an inverted fluorescence microscope at 40x magnifications.

GO orbital fibroblasts were treated with 2% CSE or 200 μM H_2_O_2_ for 30 minutes. In *PTPN1*-silenced GO cells, CSE- or H_2_O_2_-induced intracellular ROS generation was reduced significantly as detected by H_2_DCFDA (*p<0.05) (Fig 3A and 3B).

### PTP1B suppression results in an anti-fibrotic effect

**Fig 4:**
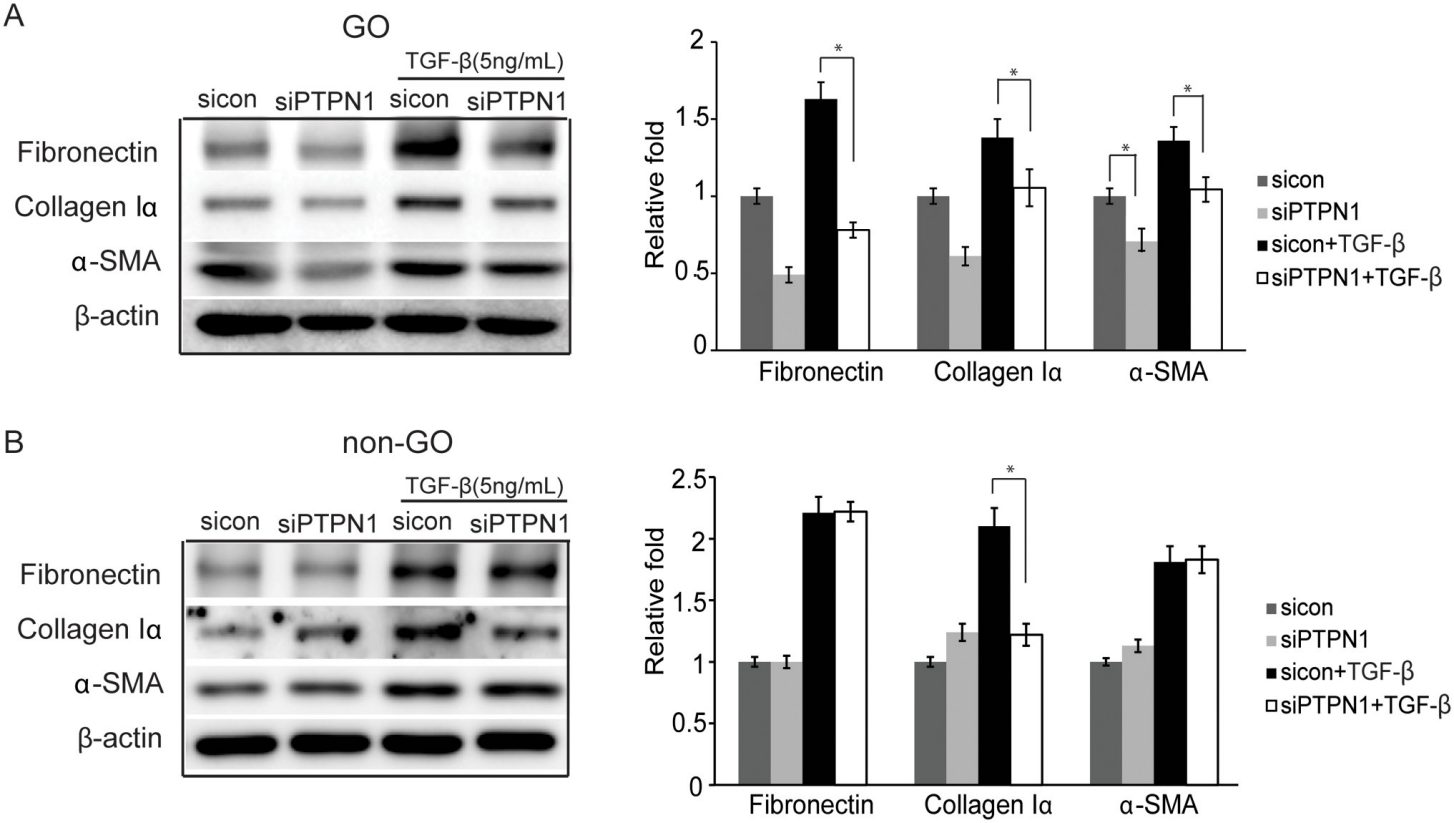
Effect of PTP1B suppression on the fibrosis of orbital fibroblasts. Orbital fibroblasts from GO and non-GO patients transfected with sicontrol or siPTPN1 for 24 hours were exposed to TGF-β (5 ng/mL) for 24 hours. Cell lysates of GO fibroblasts (A) and non-GO fibroblasts (B) were subjected to western blot analysis of fibronectin, collagen Iα, and α-SMA protein expression. Experiments were performed in three GO cell strains and non-GO cell strains taken from different individuals. Data in the columns indicate the mean density ratio ± SD by densitometry normalised to the level of β-actin in the same sample. Representative gel images are shown. Differences between sicontrol and siPTPN1 are indicated (*p<0.05). See S3 Fig for uncropped blots.

We also investigated whether PTP1B suppression has an effect on the expression of fibrosis-related proteins. Fibronectin, collagen Iα, and α-SMA protein expression was evaluated after 24 hours of TGF-β (5 ng/mL) stimulation in sicontrol cells and siPTPN1 cells. Enhanced expression of fibronectin, collagen Iα, and α-SMA following TGF-β treatment was significantly inhibited in the siPTPN1-transfected GO cells (*p<0.05) (Fig 4A). In non-GO cells, only collagen Iα was inhibited after *PTPN1* blocking (*p<0.05) (Fig 4B).

### Role of PTP1B suppression in ER stress

**Fig 5:**
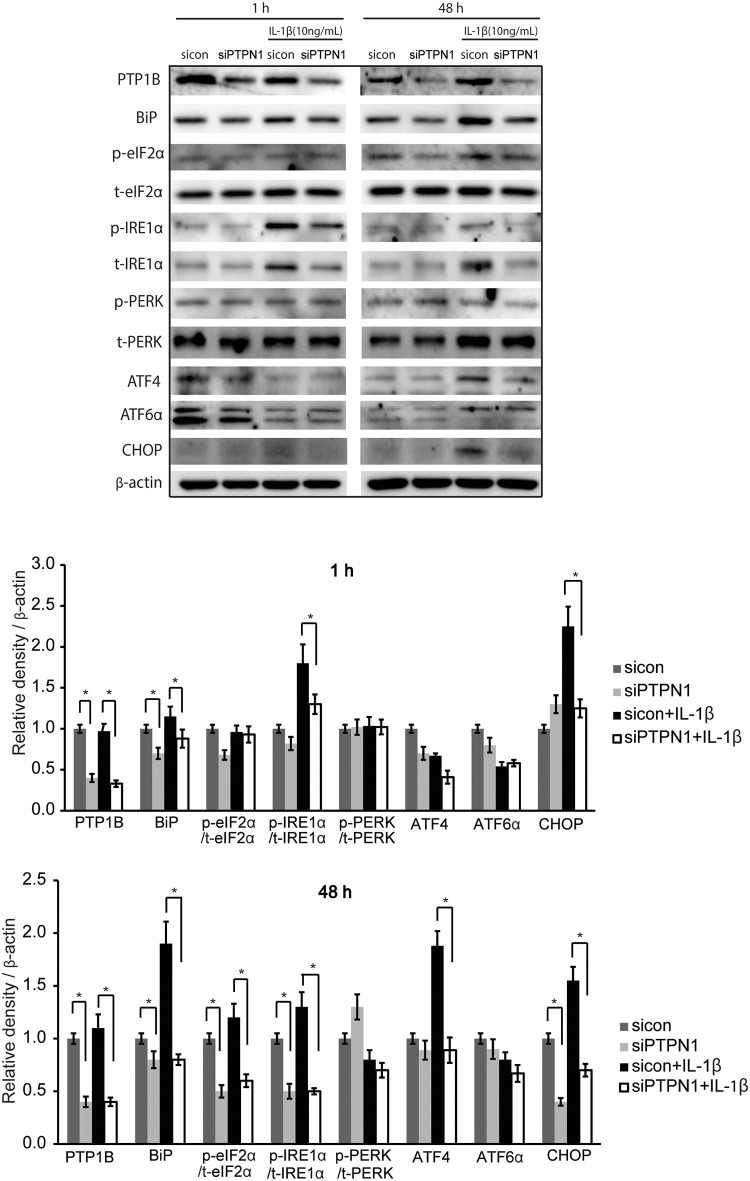
Role of PTP1B suppression on ER stress. GO fibroblasts were transfected with sicontrol or siPTPN1 for 24 hours and exposed to IL-1β (10 ng/mL) for 1 hour or 48 hours. The GO fibroblasts were lysed and proteins related ER stress such as BiP, CHOP, ATF4, ATF6α, phosphorylated and total eIF2α, IRE1α, and PERK were analysed by western blot. Experiments were performed in three GO cells from different individuals. (A) Representative gel images are shown. (B,C) Data in the columns represent the mean density ratio ± SD by densitometry normalised to the level of β-actin in the same sample for 1 hour and 48 hours, respectively. Differences between sicontrol and siPTPN1 are indicated (*p<0.05). See S3 Fig for uncropped blots.

PTP1B is a negative regulator of insulin signalling and is localised on the ER membrane. ER stress was induced in GO cells by treatment with IL-1β (10 ng/mL) for 1 hour and 48 hours. Silencing of *PTPN1* with siRNA significantly suppressed the protein expression of BiP, p-IRE1α, and CHOP induced by 1 hour IL-1β treatment as well as the protein expression of BiP, p-eIF2α, p-IRE1α, ATF4, and CHOP induced by 48 hours of IL-1β treatment in GO cells (*p<0.05). The expression levels of PERK and ATF6α were not changed by silencing of *PTPN1* (Fig 5).

We investigated the effect of *PTPN1* silencing on cell viability and apoptosis whether the suppression of ER stress protein by PTP1B suppression was associated with apoptosis [20, 21]. Upon IL-1β stimulation for 48 hours, GO fibroblasts displayed increased proliferative activity (*p<0.05). Treatment with IL-1β for 24 hours and 48 hours in both sicontrol and siPTPN1-transfected cells did not change cell viability in both GO and non-GO fibroblasts (S2A Fig). The sicontrol and siPTPN1-transfected cells were subjected to an Annexin V apoptosis assay after incubation for 24 and 48 hours. *PTPN1* inhibition did not significantly increase apoptosis or necrosis in GO and non-GO fibroblasts (S2B Fig).

### Effect of PTP1B suppression on signal pathway molecules

**Fig 6:**
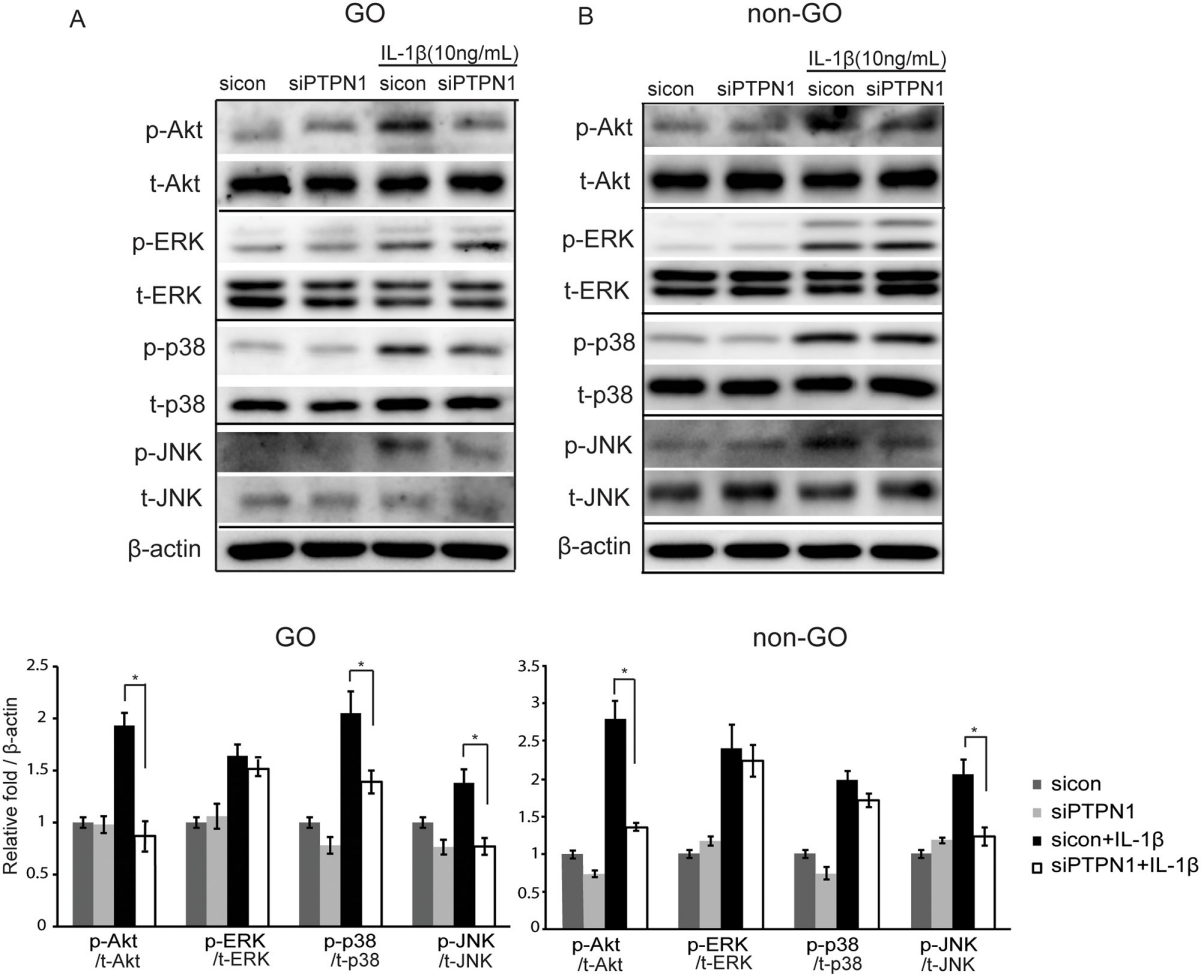
Effect of PTP1B suppression on signalling pathways in orbital fibroblasts. GO and non-GO cells were transfected with sicontrol or siPTPN1 for 24 hours and then exposed to IL-1β (10 ng/mL) for 1 hour. Phosphorylated and total Akt, ERK, p38, and JNK were determined by western blotting in GO fibroblasts (A) and non-GO fibroblasts (B). Experiments were performed in three GO and non-GO cells from different individuals. Representative bands are shown. Data in the columns indicate the mean density ratio ± SD by densitometry normalised to the level of β-actin in the same sample. Differences between sicontrol and siPTPN1 are indicated (*p<0.05). See S3 Fig for uncropped blots.

The phosphoinosidie 3-kinase (PI3K)-Akt pathway has been reported to be involved in GO [22, 23] and mitogen-activated protein kinase (MAPK) is well-known for its involvement in inflammatory diseases [24, 25]. After treatment of sicontrol fibroblasts with IL-1β (10 ng/mL) for 1 hour, Akt, ERK, p38, and JNK phosphorylation increased significantly in both GO and non-GO cells. However, when PTP1B was suppressed by siPTPN1 transfection, Akt, p38, and JNK phosphorylation was significantly reduced in GO cells (*p<0.05) (Fig 6A); Akt and JNK phosphorylation was inhibited in non-GO cells (*p<0.05) (Fig 6B).

## Discussion

In this study, we investigated the role of PTP1B in the pathogenesis of GO and evaluated whether suppression of PTP1B could reverse inflammation, oxidative stress, and fibrosis in GO cells. *PTPN1* silencing significantly inhibited proinflammatory cytokine production, ROS generation induced by H_2_O_2_ or CSE, and the expression of proteins related to TGF-β-induced fibrosis in GO cells. Furthermore, *PTPN1* silencing resulted in the amelioration of ER stress and suppression of the phosphorylation of Akt, p38, and JNK signalling pathway molecules without affecting cell viability. Protein tyrosine phosphorylation is regulated through coordination between protein tyrosine kinases and phosphatases; disturbance of the balance between the two enzymes results in aberrant tyrosine phosphorylation, which is implicated in several human diseases, including cancer, diabetes, and inflammation [26]. This study is, to our knowledge, the first to find that PTP1B suppression has a protective effect *in vitro* against GO pathologic mechanisms.

Recently, bidirectional crosstalk between TSHR and IGF-1R was demonstrated in GO fibroblasts. Here, we evaluated whether PTP1B was stimulated by M22, bTSH, IGF-1, IL-1β, or TNF-α in GO fibroblasts. There have been no previous studies linking TSHR and PTP1B signalling. In this study, we demonstrated that the treatment of orbital fibroblasts with TSHR or IGFR ligands upregulated the expression of PTP1B in both GO and non-GO cultures, although the mechanism is still unclear. IGF-1 activity is reported to be negatively controlled by the action of PTP1B [27] and inhibiting PTP1B is considered to have neuroprotective effects in diabetic retinopathy by modulating IGF-1 activity [28]. Proinflammatory cytokines have previously been shown to increase PTP1B levels, similar to our findings in orbital fibroblasts. TNF-α treatment increased PTP1B levels by 2- to 5-fold in adipocytes and hepatocytes; moreover, administration of TNF-α to mice resulted in elevated PTP1B mRNA levels in the adipose tissue, skeletal muscle, hypothalamic arcuate nucleus, and liver [29].

Orbital inflammation progresses by the infiltration of mononuclear cells, such as B-lymphocytes, macrophages, and especially T-lymphocytes, into the orbital tissue in GO [30, 31]. Furthermore, as previously reported [32], GO fibroblast was more inflammatory by nature and were sensitive to stimulation. Herein, compared to non-GO cells, GO fibroblasts displayed increased secretion of pro-inflammatory cytokines upon IL-1β stimulation and even in naïve conditions. *PTPN1* silencing in GO cells reduced proinflammatory proteins, including IL-6, IL-8, MCP-1, Cox-2, ICAM1, and p-NF-κB. However, in non-GO fibroblasts, proinflammatory proteins except for Cox-2 were not significantly curtailed upon PTP1B suppression. PTP1B seems to play a specific role in GO cells, which serves as a positive regulator of inflammation.

There are still controversies regarding the role of PTP1B in the inflammatory mechanism. It has been shown that PTP1B plays a key role in converting dendritic cells to an active and mature state and activating T cells by displaying podosomes [33]. In PTP1B-deficient mice with induced colitis, inflammation was suppressed by the production of myeloid-derived suppressor cells, which have characteristic T cell-suppressant functions [34]. PTP1B blockage in microglial cells inhibited the production of lipopolysaccharide-induced proinflammatory molecules, such as IL-1β, TNF-α, Cox-2, and iNOS [35], and protected microglial cells against hypothalamic inflammation by activating the JAK2-STAT3 signalling pathway [36]. In contrast, there are several opposing reports insisting that PTP1B is a negative regulator of inflammation. PTP1B knockout in the spleen leukocytes of mice resulted in increased chemotaxis and transendothelial migration and exacerbated allergic inflammation [37]. Acute pancreatitis was enhanced in PTP1B knockout mice via increased production of inflammatory cytokines, such as IL-1β, IL-6, and TNF-α [38]. PTP1B is ubiquitously expressed and performs different functions in various tissues. Therefore, modulation of PTP1B has different results depending on the cell type *in vitro* as well as the specific tissue *in vivo* [6, 11].

PTP1B has been reported to promote fibrosis in many other cell types. Genetic deletion of PTP1B and administration of PTP1B inhibitors reduced cardiac fibrosis in chronic heart failure mice [39]. In the endothelial cell-specific PTP1B KO mouse, cardiac fibrosis was reduced via elevation in caveolin-1, which inhibited ROS generation and suppressed TGF-β signalling [40]. In addition, PTP1B plays a regulatory role in liver fibrosis. PTP1B blocking using siRNA inhibited the proliferation and activation of hepatic stellate cells induced by TGF-β1 [41]. PTP1B-depleted hepatocytes showed resistance to TGF-β through the activation of NADPH oxidase 1 [42]; moreover, hepatic fibrosis in response to cholestatic liver injury was ameliorated by the same mechanism [43]. In our study on GO orbital fibroblasts, *PTPN1* silencing down-regulated all fibrosis-related proteins induced by TGF-β, which was not evident in non-GO fibroblasts.

Systemic hyperthyroidism and T cell infiltration into orbital tissue leads to ROS production, and oxidative stress from cigarette smoking can aggravate GO severity by increasing T cell proliferation, adipogenesis, and glycosaminoglycan production in orbital fibroblasts [44–46]. Our study shows that *PTPN1* silencing ameliorated ROS generation in both CSE- and H_2_O_2_-stimulated cells. Regarding oxidative stress, PTP1B deficiency in skeletal muscle cells resulted in the reduction of ceramide and ROS levels, whereas overexpression of PTP1B showed the opposite effect [47]. PTP1B deletion reduced mitochondrial dysfunction by increasing the expression of SIRT1 and reducing the phosphorylation of p65. PTP1B inhibition in mouse hepatocytes ameliorated acetaminophen-induced cell death and reduced GSH depletion, ROS generation, and activation of JNK and p38. PTP1B inhibition also enhanced the expression of the Nrf2-target gene heme oxygenase-1 (HO-1), suggesting that PTP1B plays a key regulatory role in the antioxidant system [48].

The changes in the phosphorylation level of multiple transcription factors following PTP1B inhibition in GO fibroblasts indicate that a more complex network of signalling pathways may exist. Kumar et al. discovered that an autoantibody to TSHR stimulated the PI3K/Akt pathway downstream and induced adipogenesis in orbital preadipocytes in GO [22]. Herein, PTP1B inhibition suppressed IL-1β-induced Akt, JNK phosphorylation in GO and non-GO fibroblasts. However, p38 MAPK phosphorylation was reduced by PTP1B inhibition only in GO fibroblasts. The p38 and JNK pathways, along with the MAPK pathway, are well-known for mediating the transcription and translation of inflammatory cytokines; moreover, they have been considered as targets of anti-inflammatory therapy [24, 25]. In several other disease-specific cell models, JNK and p38 phosphorylation was ameliorated by PTP1B inhibition [47, 48].

The downregulation of inflammation and its associated transcription factors with siPTPN1 may be related to a blocked ER stress response. It has been well-documented that the ER stress response, also known as the unfolded protein response (UPR), activates inflammation by inducing the NF-κB and JNK pathways [49, 50]. The upregulation of UPR molecules in autoimmune diseases, such as rheumatoid arthritis, systemic lupus erythematosus, and inflammatory bowel syndrome, has shown this relationship [50, 51]. PTP1B is located in the cytoplasmic region of the ER and plays an active role in the UPR. Gu et al. reported that PTP1B KO embryonic fibroblasts showed reduced IRE1 signalling, specific XBP-1 splicing, and JNK pathway activation; moreover, they were resistant to ER stress-induced apoptosis [52]. Similarly, liver-specific PTP1B deletion impaired the UPR by suppressing the phosphorylation of UPR molecules such as PERK, eIF2α, p38, JNK, and CHOP under a high fat-induced ER stress environment [53, 54]. Additionally, mouse myoblasts transfected with PTP1B siRNA exhibited attenuated phosphorylation of eIF2α and JNK1/2 [55]. Our results demonstrated that *PTPN1* silencing blocks part of the UPR cascade by reducing the expression of proteins such as BiP, eIF2α, IRE1α, and CHOP. However, *PTPN1* silencing did not affect apoptosis and cell viability.

To our knowledge, this study demonstrates for the first time that PTP1B mediates inflammatory reactions in GO fibroblasts. We found that *PTPN1* silencing attenuated inflammation, fibrosis, and ROS generation, which could possibly be associated with the suppression of the ER stress response pathway and Akt, JNK, and p38 phosphorylation in GO cells. Inhibiting PTP1B might be a novel therapeutic option for the treatment of GO related to inflammatory and oxidative stressors. In addition, the inhibitory effect of PTP1B modulation on TGF-β induced fibrosis could also contribute to controlling fibrotic events in GO. Further studies are necessary to prove the *in vivo* effect of PTP1B inhibition in GO in clinically applications.

## Supporting information

S1 Fig TLR expressions depending on *PTPN1* silencing in orbital fibroblasts. GO and non-GO fibroblasts were transfected with sicontrol or siPTPN1 for 24hours. TLR2, 4, and 9 expression levels were quantified with RT-PCR. Data in the columns indicate relative TLR mRNA fold of siPTPN1 cells normalised to sicontrol of each cell type (*p<0.05, sicontrol versus siPTPN1). Experiments were performed in three GO and non-GO fibroblasts from different individuals.
(TIF)

S2 Fig Effect of PTP1B blocking on cell viability and apoptosis. Orbital fibroblasts from GO and non-GO patients were transfected with sicontrol and siPTPN1 for 24 hours. (A) After the transfection, cells were cultured in different conditions (with or without IL-1β) for 24 hours and 48 hours. Then, the viability of the cells was assessed with MTT assay. Difference between sicontrol and sicontrol with IL-1β stimulation in GO was indicated (*p<0.05). (B) The transfected cells were subjected to Annexin V apoptosis assay after incubating for 24 hours and 48 hours. Assays were performed using three GO and non-GO cells from different individuals.
(TIF)

S3 Fig Uncropped version of western blots. (TIF)
