# Supplementary figures and images for: Protein tyrosine phosphatase 1B as a therapeutic target for Graves’ orbitopathy in an in vitro model
Source: PLoS One. 2020 Aug 6;15(8):e0237015. doi: 10.1371/journal.pone.0237015 (PMC7410323; doi:10.1371/journal.pone.0237015)

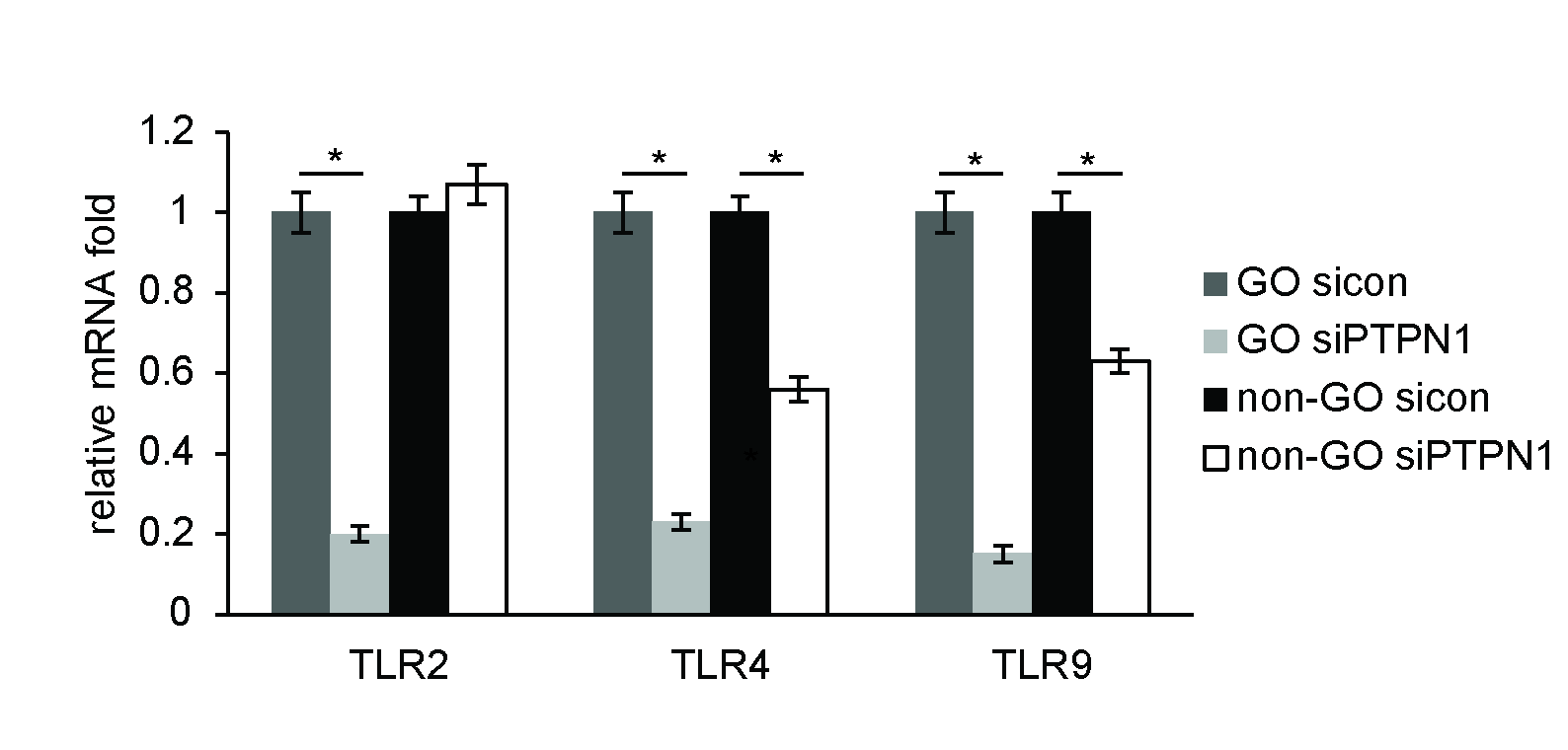

Supplement: S1 Fig — GO and non-GO fibroblasts were transfected with sicontrol or siPTPN1 for 24hours. TLR2, 4, and 9 expression levels were quantified with RT-PCR. Data in the columns indicate relative TLR mRNA fold of siPTPN1 cells normalised to sicontrol of each cell type (*p<0.05, sicontrol versus siPTPN1). Experiments were performed in three GO and non-GO fibroblasts from different individuals. (TIF) [file pone.0237015.s001.tif]

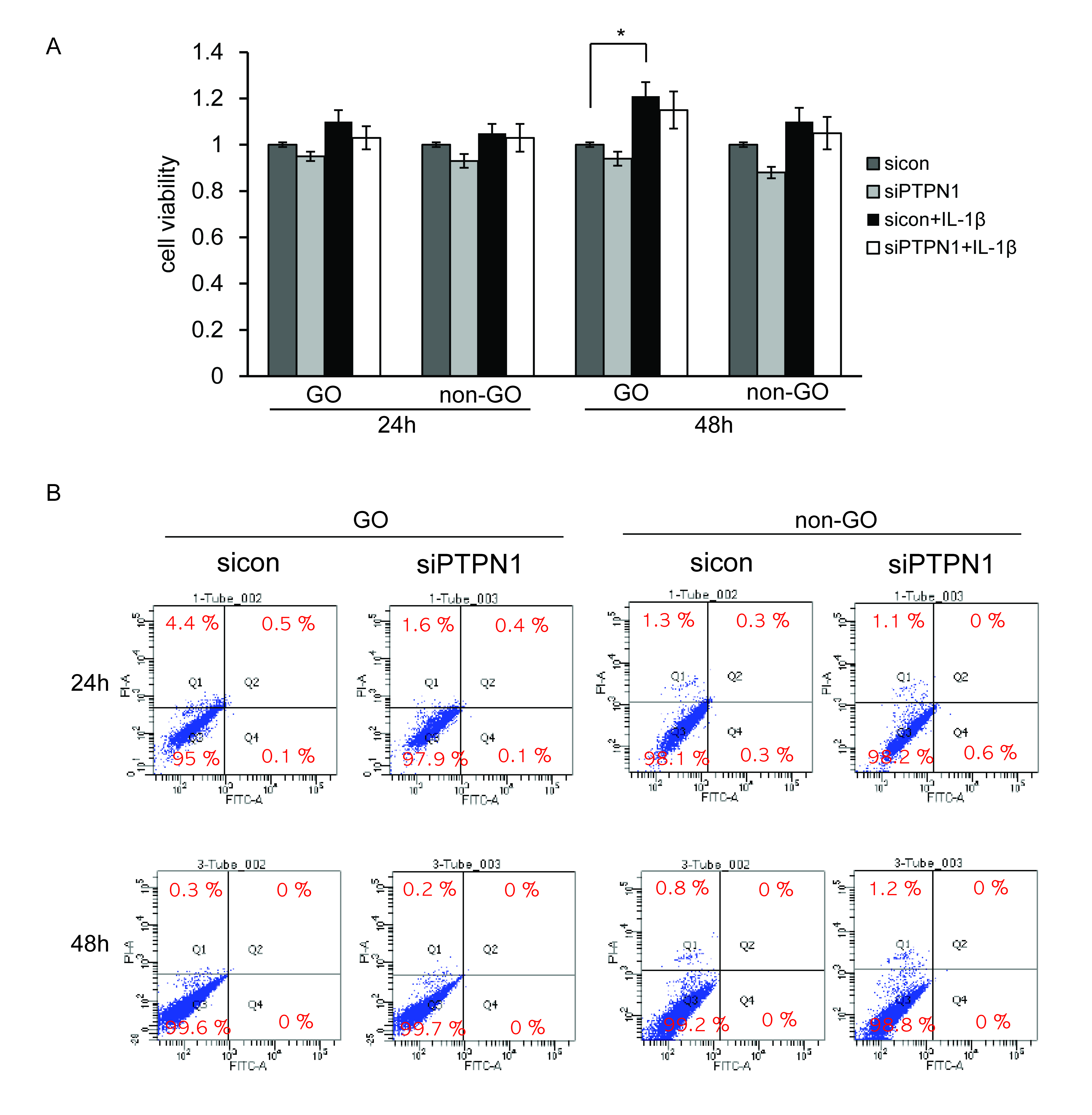

Supplement: S2 Fig — Orbital fibroblasts from GO and non-GO patients were transfected with sicontrol and siPTPN1 for 24 hours. (A) After the transfection, cells were cultured in different conditions (with or without IL-1β) for 24 hours and 48 hours. Then, the viability of the cells was assessed with MTT assay. Difference between sicontrol and sicontrol with IL-1β stimulation in GO was indicated (*p<0.05). (B) The transfected cells were subjected to Annexin V apoptosis assay after incubating for 24 hours and 48 hours. Assays were performed using three GO and non-GO cells from different individuals. (TIF) [file pone.0237015.s002.tif]

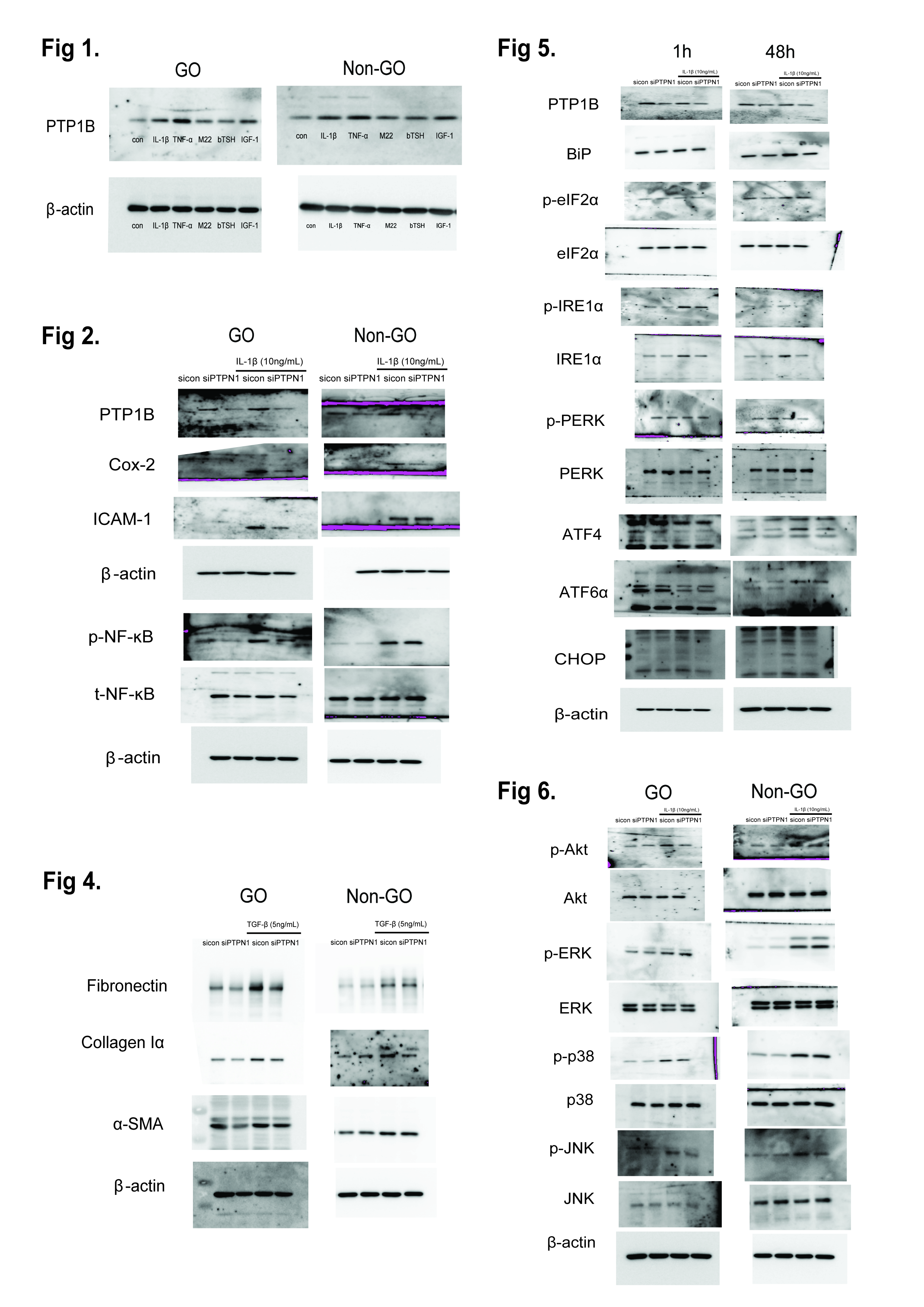

Supplement: S3 Fig — (TIF) [file pone.0237015.s003.tif]
